# Supplementary material for: Modeling glioblastoma heterogeneity as a dynamic network of cell states
Source: Mol Syst Biol. 2021 Sep 16;17(9):e10105. doi: 10.15252/msb.202010105 (PMC8444284; doi:10.15252/msb.202010105)
Supplement: Supplementary file 6 — Source Data for Figure 5 [file MSB-17-e10105-s004.zip › Figure5A_sourcedata/GSEA_3017/nosigdb_stateF.GseaPreranked.1622795015644/ALLEN_113_ASTROL1FGFR3FOS.html]

Details for gene set ALLEN\_113\_ASTROL1FGFR3FOS[GSEA]

|  || Dataset | state33017 |
| Phenotype | NoPhenotypeAvailable |
| Upregulated in class | na\_pos |
| GeneSet | ALLEN\_113\_ASTROL1FGFR3FOS |
| Enrichment Score (ES) | 0.73981684 |
| Normalized Enrichment Score (NES) | 2.825803 |
| Nominal p-value | 0.0 |
| FDR q-value | 0.0 |
| FWER p-Value | 0.0 |
Table: GSEA Results Summary

  

Fig 1: Enrichment plot: ALLEN\_113\_ASTROL1FGFR3FOS      
 Profile of the Running ES Score & Positions of GeneSet Members on the Rank Ordered List

  

| PROBE | GENE SYMBOL | GENE\_TITLE | RANK IN GENE LIST | RANK METRIC SCORE | RUNNING ES | CORE ENRICHMENT || 1 | NEAT1 |  |  | 0 | 2.864 | 0.1951 | Yes |
| 2 | CST3 |  |  | 15 | 1.273 | 0.2755 | Yes |
| 3 | S100B |  |  | 20 | 1.167 | 0.3533 | Yes |
| 4 | SLC1A3 |  |  | 26 | 1.058 | 0.4231 | Yes |
| 5 | SLC4A4 |  |  | 40 | 0.980 | 0.4840 | Yes |
| 6 | GFAP |  |  | 47 | 0.953 | 0.5463 | Yes |
| 7 | AQP4 |  |  | 84 | 0.789 | 0.5839 | Yes |
| 8 | CRYAB |  |  | 85 | 0.783 | 0.6372 | Yes |
| 9 | ADGRV1 |  |  | 86 | 0.782 | 0.6905 | Yes |
| 10 | PON2 |  |  | 93 | 0.764 | 0.7398 | Yes |
| 11 | ITPKB |  |  | 279 | 0.525 | 0.6928 | No |
| 12 | ID2 |  |  | 366 | 0.483 | 0.6872 | No |
| 13 | FGFR3 |  |  | 515 | 0.429 | 0.6502 | No |
| 14 | ID4 |  |  | 659 | 0.383 | 0.6123 | No |
| 15 | NKAIN3 |  |  | 765 | 0.354 | 0.5894 | No |
| 16 | SLC7A11 |  |  | 1131 | 0.288 | 0.4457 | No |
| 17 | NCKAP5 |  |  | 1279 | 0.271 | 0.3984 | No |
| 18 | KANK1 |  |  | 1449 | 0.252 | 0.3399 | No |
| 19 | ZFP36L1 |  |  | 1590 | -0.284 | 0.2966 | No |
Table: GSEA details [plain text format]

  

Fig 2: ALLEN\_113\_ASTROL1FGFR3FOS: Random ES distribution      
 Gene set null distribution of ES for **ALLEN\_113\_ASTROL1FGFR3FOS**
